# Predictors of recognition of out of hospital cardiac arrest by emergency medical services call handlers in England: a mixed methods diagnostic accuracy study

**DOI:** 10.1186/s13049-020-00823-9

**Published:** 2021-01-06

**Authors:** Caroline L. Watkins, Stephanie P. Jones, Margaret A. Hurley, Valerio Benedetto, Christopher I. Price, Christopher J. Sutton, Tom Quinn, Munirah Bangee, Brigit Chesworth, Colette Miller, Dawn Doran, Aloysius Niroshan Siriwardena, Josephine M. E. Gibson

**Affiliations:** 1 School of Nursing, University of Central Lancashire, Brook Building, Preston, PR1 2HE UK; 2 Newcastle University, Newcastle, UK; 3 The University of Manchester, Manchester, UK; 4 Kingston University London and St George’s, University of London, London, UK; 5 University of Lincoln, Lincoln, UK

**Keywords:** Out-of-hospital cardiac arrest, Emergency medical dispatch, Diagnostic accuracy, Symptom recognition

## Abstract

**Background:**

The aim of this study was to identify key indicator symptoms and patient factors associated with correct out of hospital cardiac arrest (OHCA) dispatch allocation. In previous studies, from 3% to 62% of OHCAs are not recognised by Emergency Medical Service call handlers, resulting in delayed arrival at scene.

**Methods:**

Retrospective, mixed methods study including all suspected or confirmed OHCA patients transferred to one acute hospital from its associated regional Emergency Medical Service in England from 1/7/2013 to 30/6/2014. Emergency Medical Service and hospital data, including voice recordings of EMS calls, were analysed to identify predictors of recognition of OHCA by call handlers. Logistic regression was used to explore the role of the most frequently occurring (key) indicator symptoms and characteristics in predicting a correct dispatch for patients with OHCA.

**Results:**

A total of 39,136 dispatches were made which resulted in transfer to the hospital within the study period, including 184 patients with OHCA. The use of the term ‘Unconscious’ plus one or more of symptoms ‘Not breathing/Ineffective breathing/Noisy breathing’ occurred in 79.8% of all OHCAs, but only 72.8% of OHCAs were correctly dispatched as such. ‘Not breathing’ was associated with recognition of OHCA by call handlers (Odds Ratio (OR) 3.76). The presence of key indicator symptoms ‘Breathing’ (OR 0.29), ‘Reduced or fluctuating level of consciousness’ (OR 0.24), abnormal pulse/heart rate (OR 0.26) and the characteristic ‘Female patient’ (OR 0.40) were associated with lack of recognition of OHCA by call handlers (*p*-values < 0.05).

**Conclusions:**

There is a small proportion of calls in which cardiac arrest indicators are described but the call is not dispatched as such. Stricter adherence to dispatch protocols may improve call handlers’ OHCA recognition. The existing dispatch protocol would not be improved by the addition of further terms as this would be at the expense of dispatch specificity.

**Supplementary Information:**

The online version contains supplementary material available at 10.1186/s13049-020-00823-9.

## Background

Non-traumatic out-of-hospital cardiac arrest (OHCA) is a leading cause of death worldwide [1–3] with an estimated incidence of 1/2000 person-years [4–6]. Typical survival rates are around 7% [7] and have not changed substantially in three decades [8]. Early effective cardiopulmonary resuscitation (CPR), early defibrillation where indicated, and rapid attendance by Emergency Medical Services (EMS) are essential to increasing the chances of survival. Fast and accurate recognition of OHCA by EMS call handlers is essential.

EMS call handlers have three roles in OHCA: 1) recognition; 2) facilitation of rapid EMS attendance; and 3) provision of bystander resuscitation instructions including identification of public access defibrillators. In many countries’ EMS services, these tasks are guided by use of computer algorithms to categorise and prioritise calls. Effective use of these algorithms relies on the call handler skilfully interpreting the caller’s description of symptoms, but this is a challenging task. A systematic review of studies of call handler-caller interaction suggests that the most sensitive and specific combination of reported symptoms of OHCA is unconsciousness together with absent or abnormal breathing, and that OHCA should also be considered if a generalised seizure is described [9], although the presence of seizure symptoms may be misinterpreted. However, ineffective or agonal breathing is present in around 40% of OHCAs [10], and can make it difficult to obtain an accurate description of the patient’s condition [9], yet its presence (rather than absent breathing) may be more likely to indicate a potentially recoverable OHCA [11, 12]. Because of these challenges, call handlers’ recognition of OHCA across studies of caller-call handler interaction ranges from 38 to 97% sensitivity [9]. These factors can give rise to delays in response, leading to poor rates of provision of bystander resuscitation instructions [13], and to reduced survival rates.

Survival to hospital discharge from OHCA in England is 8.6% (range 2.2–12.0%) [14, 15], while survival rates of up to 25% have been reported in some other countries, albeit for selected patient cohorts [16]. A study in Northern Ireland demonstrated that a contributing factor to low survival rates was poor call handler sensitivity (< 70%) to identification of OHCA [17]. Furthermore, EMS staff typically make a resuscitation attempt in fewer than 50% of OHCAs they attend [18–21], often when pre-arrival delay or inaction renders it futile. However, attempts to increase sensitivity may lead to reduced specificity and to inappropriate OHCA dispatch for non-OHCA calls. In England in 2017, approximately 150,000 ambulance calls (2.2% of all calls for which a face-to-face response was provided) were dispatched with the highest level of response (Category 1) which is required where there is an immediate threat to life, of which one in five proved to be an OHCA requiring on-scene resuscitation [22]. Better specificity could therefore improve resource use, but it is unclear how to achieve this without compromising sensitivity or speed of response.

The aim of this study was to explore predictors of recognition of OHCA by EMS call handlers in England.

## Objectives

1. To make estimates of the diagnostic test accuracy of the ‘key indicator symptoms’ which are indicative of people in cardiac arrest;
2. To synthesise the findings from the identification of the ‘key indicator symptoms’ and variables predictive of cardiac arrest, to determine if it is possible to improve recognition of OHCA

## Methods

This was a retrospective study using qualitative and quantitative methodologies.

### Setting

The setting was one EMS in England receiving 1.3 million calls per year, and one acute hospital within this EMS region covering a catchment population of 370,000. In England, calls to the EMS are triaged using one of two systems, the Advanced Medical Priority Dispatch System (AMPDS) or NHS Pathways. The OHCA dispatch protocol for this study was version 12.1 of AMPDS. AMPDS, an algorithm-based system which is used widely in Europe and North America, is designed to standardise call handler practice, and to ensure safe and effective patient care. The AMPDS system prompts the call handler with an on-screen message to ask: “What’s the problem, tell me exactly what happened,’ followed by the further questions: ‘Is s/he conscious?’ and ‘Is s/he breathing?’ If the answers are negative to the last two questions, the dispatcher assigns a cardiac arrest code to the incident, necessitating the highest level of EMS response.

### Subjects and sampling

Cases, occurring from 1st July 2013 to 30th June 2014, were eligible for inclusion if they were patients of any age with: 1) Suspected cardiac arrest (CA) or respiratory arrest for whom an ambulance was dispatched in response to an emergency call, resulting in the patient being transferred to the study hospital; or 2) identified as having had CA from hospital and ambulance records but whose CA was unrecognised at the point of ambulance dispatch. Cases were excluded if the patient was not transferred to the study hospital (i.e. those who were transported to another hospital or where the patient was recognised as having died on scene). Patients were identified either from the EMS and matched with their hospital records, or, conversely, from hospital records matched with their EMS records. Matching was performed by researchers at the EMS and the hospital, principally by dispatch incident number.

### Definition of OHCA

The AMPDS algorithm for all calls asks if the patient is conscious and breathing. If the patient is reported to be unconscious and either not breathing or breathing abnormally, the call should be coded by the dispatcher as a cardiac arrest. The actual occurrence of CA was determined by the final diagnosis in hospital and by reference to the EMS patient report form (EPRF). The definition of CA included documented respiratory arrest [19]. Cases of CA were then divided into two types: those which were confirmed as OHCA and those which did not qualify as OHCA. We classified cases as OHCA based on the EPRF, including free text documentation, where the person had been found to be in cardiac arrest at the time of arrival of EMS personnel at the scene. We also included cases where there was clear documentation in the EPRF of the deployment of a public automated external defibrillator with return of spontaneous circulation prior to the arrival of EMS personnel. In either of these situations it was not possible for us to verify exactly when the cardiac arrest occurred, and in particular whether it had occurred before or during the EMS call, only that it had occurred prior to the arrival of EMS personnel on scene. The focus of this study was dispatcher identification of OHCA during the initial EMS call, corresponding to Utstein data definition ‘Dispatcher identified presence of cardiac arrest’; the further Utstein characteristics ‘Dispatcher provided CPR instructions’, plus patient age and gender were also of relevance [23]. We therefore excluded cases from the definition of OHCA where the person had a CA only after the arrival of EMS personnel at the scene. We also excluded cases where the patient was conveyed to a different hospital or was not conveyed.

### Classification of incidents

True positive: (TP): Cases which were dispatched by the EMS as cardiac arrest and where there was a subsequent diagnosis by the EMS and/or hospital of cardiac arrest, occurring before the arrival of EMS.

False Positive (FP): Cases which were dispatched by the EMS as cardiac arrest but where there was no subsequent diagnosis by either the EMS or hospital of cardiac arrest, or where cardiac arrest occurred subsequent to the arrival of EMS.

False Negative (FN): Cases which were not dispatched by the EMS as cardiac arrest but where there was a subsequent diagnosis of cardiac arrest having occurred before the arrival of EMS.

True Negative (TN): Cases which were not dispatched by the EMS as cardiac arrest and where there was no subsequent diagnosis by the EMS or hospital of pre-arrival cardiac arrest.

### Analysis of voice recordings

All voice recordings of EMS calls were listened to in full and qualitative analysis performed with blinding to the categorisation of the call (by JG, MB, BC, DD, SJ, and CM) for clinically relevant content about all symptoms reported by the caller. Verbatim accounts of symptoms provided by callers were then clustered using an iterative process to generate indicator symptoms: for example, verbatim descriptions such as ‘I can’t tell if he’s breathing’; ‘she’s breathing on and off’; ‘taking the odd breath’; ‘struggling to breathe’; were clustered into the indicator symptom ‘Ineffective breathing’. In order to ensure that all researchers coded the call content in the same way, the first 50 calls were coded by all members of the research team and any discrepancies were discussed and resolved. Thereafter, every 10th call was coded by two members of the team. Symptoms reported by the callers during the entirety of the call were coded. This data was then quantified to determine the number and characteristics of calls in which each indicator symptom occurred.

### Data analysis

All dispatch incidents from the EMS where the patient was transferred to hospital within the timeframe of the study were enumerated and sorted by dispatch code. The dispatch codes which had been allocated by call handlers in the TP and FN cases were identified and used to select the sub-population of all incidents with these codes. This sub-population represented cases where OHCA could have plausibly occurred or been suspected, in order to provide a better basis for estimating realistic specificity than if the population of all incidents was used, and to avoid the problem of specificity being estimated unrealistically at virtually 100%. The TN cases in the sub-population were identified and then randomly sampled using dispatch code as the sampling strata and with a sampling fraction of 1.28%. This fraction was chosen so that sensitivity and specificity would have similar precision of estimation.

After computation of the diagnostic test accuracy (DTA) measures, key indicator symptoms were ranked by sensitivity and compared to call handlers’ performance. Combinations of key indicator symptoms were then explored to see which might outperform call handler practice, via better sensitivity or specificity.

In addition, logistic regression explored the role of the key indicator symptoms, together with patient age and sex, in predicting a correct dispatch for patients who had OHCA. Unadjusted analysis was used first and those variables with a *p*-value less than 0.2, indicating that a variable was potentially predictive, were selected and then entered into an adjusted analysis. Those variables which still retained a p-value of less than 0.2 in the adjusted analysis were retained in a final minimal model.

We used the Standards for Reporting Diagnostic Accuracy Studies (STARD) guidelines for reporting DTA studies where possible [24].

### Patient and public involvement

Patients and public involvement representatives contributed to the design of the study, project management and steering group meetings, the writing of lay documents and summaries. Permission was granted by the Confidentiality Advisory Group for the use of non-anonymised voice files of emergency ambulance calls. Therefore, dissemination to individual study participants was not possible.

## Results

Of 39,136 dispatches made by the EMS to the hospital during the study period, 184 patients with OHCA were identified, of whom 134 were correctly dispatched and were classified as TP cases (see Table 1). A further 74 FP dispatches were identified. A total of 14,227 incidents occurred in the sub-population using the dispatch codes recorded for the TP and FN cases. These results estimated a sensitivity of 72.8% (CI 65.8 to 79.1%) and a specificity of 99.4% (CI 99.3 to 99.6%) for call handlers’ recognition of OHCA.

**Table 1:** Estimated sensitivity and specificity of individual key indicator symptoms reported in EMS calls, compared to call handlers’ performance

	Sensitivity	(95% CI)	Specificity	(95% CI)
**Call handlers’ performance**	**72.8**	**(65.8–79.1)**	**99.4**	**(99.3–99.6)**
**Key indicator symptom**				
Unconscious	86.0	(80.2–90.5)	93.4	(89.0–96.2)
Not Breathing	57.9	(50.6–65.0)	97.5	(94.4–99.1)
Ineffective Breathing	57.3	(50.0–64.5)	60.5	(53.2–67.4)
Collapse	43.8	(36.6–51.1)	84.4	(78.8–89.2)
Breathing Yes/Effective	30.3	(23.9–37.6)	16.4	(11.8–22.6)
Change in Colour	29.2	(22.9–36.0)	81.7	(75.6–86.8)
Noisy Breathing	25.8	(19.8–32.8)	92.2	(87.4–95.3)
Died	21.9	(16.2–28.6)	99.3	(96.9–99.9)
Mouth/Vomit	18.5	(13.4–24.9)	89.4	(84.2–93.2)
Conscious	16.3	(11.2–22.4)	19.8	(14.3–26.0)
Eyes open/Staring	15.7	(11.0–21.8)	97.1	(93.9–98.8)
Cool/Clammy/Cold	15.7	(10.7–21.6)	77.9	(71.3–83.6)
Reduced or Fluctuating Consciousness	15.2	(10.3–20.9)	79.6	(73.2–84.9)
Sudden Onset/Deterioration	13.5	(9.0–19.1)	96.1	(92.4–98.3)
Major Trauma/Haemorrhage	13.5	(8.9–19.2)	95.5	(91.6–97.8)
Serious/Urgent Problem	10.1	(6.3–15.6)	97.2	(93.9–98.8)
Cardiac Symptoms/Disease^1^	7.9	(4.4–12.4)	85.1	(79.5–89.9)
No Pulse/Output	7.3	(4.0–11.7)	99.4	(97.1–100)
Bystander resuscitation^2^	6.7	(3.6–11.0)	100.0	(99.9–100)
Uncoordinated Movement	6.7	(3.7–11.2)	97.2	(93.7–98.8)
Seizure-like Activity	6.7	(3.6–11.1)	98.3	(95.4–99.4)
Unknown or Non-specific Problem	5.1	(2.4–9.1)	92.3	(87.6–95.6)
Abnormal Pulse/Heart Rate	3.4	(1.2–6.8)	97.8	(94.5–99.4)
Stroke-like Symptoms	2.8	(1.1–6.2)	96.1	(92.2–98.3)
Self-harm	2.2	(0.6–5.5)	99.4	(96.8–100)
Psychiatric Symptoms	1.7	(0.5–4.4)	97.2	(94.0–98.9)
Death Imminent	0.6	(0.0–2.7)	99.5	(97.0–100)
Drug/Alcohol use	0.6	(0.0–2.8)	93.9	(89.6–96.8)

^1^e.g. chest pain, medical history, ^2^Including Airway Management /Defibrillation use

Mean age for the OHCA samples was similar for TP (63.8 years) and FN (62.3 years) groups. Mean age was slightly higher in females compared to males in the TP group (67.9 versus 63.9 years). However, females were significantly older than males in the FN group (72.2 versus 60.8 years). There was a higher proportion of male patients in the TP group than the FN group (69.2% versus 58.0%). There was no significant difference between the TP and FN groups in rates of survival to hospital discharge (33.1% TP group versus 26.0% FN group), as although survival rates were higher in the TP group, the sample sizes were small.

Two hundred and ninety eight voice calls were listened to across the TP/FP/FN groups, only 17 voice calls being unavailable. A sample of 181 voice calls was listened to from the 14,112 TN dispatches (sampling fraction, f = 181/14112 = 0.0128). In 12 of the 50 FN calls at step 2, CPR instructions were given at some time during the call, indicating that the call handler had recognised OHCA but only after a dispatch code had been assigned. Twenty-eight distinct key indicator symptoms were identified from the calls. These symptoms were ranked by sensitivity and compared to call handlers’ performance (Table 1). The individual symptom ‘Unconscious’ had higher sensitivity but lower specificity, while the terms ‘Bystander resuscitation/Airway management/Defibrillation use’, and ‘Death imminent’, had better specificity but much poorer sensitivity.

Full compliance with the EMS protocol should lead to a ‘cardiac arrest’ dispatch in all cases where the patient is described as both unconscious and with absent, noisy, or ineffective breathing. The DTA measures for the symptom ‘Unconscious’, combined with one or more of the symptoms, ‘Ineffective Breathing’, ‘Not Breathing’ or ‘Noisy Breathing’ were therefore compared with call handlers’ performance (Table 2). There was a 7% difference (CI − 1.8 to 15.7%) in sensitivity between the combined occurrence of these key indicator symptoms (79.8%) and call handlers’ performance (72.8%). For all TP, FP and FN calls, CPR was initiated independently by a bystander in 10.4% of cases. CPR instructions were given by the call-handler in a further 40.0% of TP/FP/FN calls.

**Table 2:** Estimated DTA measures (95% CI) of the key indicator symptom combinations Unconscious, alone and with any one or more symptoms: Not Breathing, Ineffective Breathing or Noisy Breathing

	Sensitivity	Specificity	PPV	NPV
Unconscious	86.0 (80.2–90.5)	93.4 (89.0–96.2)	14.0 (8.9–22.7)	99.8 (99.7–99.9)
Unconscious and (A) Noisy Breathing	23.6 (17.8–30.4)	98.8 (96.1–99.9)	19.3 (6.7–67.2)	99.0 (98.9–99.2)
Unconscious and (B) Ineffective Breathing	51.7 (44.3–58.9)	97.4 (94.1–99.0)	20.0 (9.7–40.2)	99.4 (99.3–99.5)
Unconscious and (C) Not Breathing	53.4 (45.8–60.5)	98.6 (95.9–99.7)	33.0 (13.4–71.6)	99.4 (99.3–99.5)
Unconscious and at least one of				
(B) Ineffective Breathing or (C) Not breathing	78.1 (71.4–83.8)	96.2 (92.8–98.2)	20.5 (11.6–35.4)	99.7 (99.6–99.8)
Unconscious and at least one of (A) Noisy Breathing,				
(B) Ineffective Breathing or (C) Not Breathing	79.8 (73.5–85.3)	95.7 (92.1–97.8)	18.7 (11.0–32.0)	99.7 (99.6–99.8)

We also explored the key indicator symptoms and demographic parameters which were associated with the correct dispatch for OHCA. Odds ratios (OR) for all key indicator symptoms, together with patient age and sex, in an unadjusted analysis can be found in Supplementary Table 3. All variables (*p* < 0.2) were taken forward to an adjusted analysis, and those which retained a *p* < 0.2 were included in a minimal model (Table 3). This showed that the symptoms ‘Breathing Yes/Effective’, ‘Abnormal Pulse/Heart Rate’, ‘Reduced or Fluctuating Consciousness Level’, or the patient being female, each reduced the likelihood that call handlers would recognise OHCA. In contrast, the presence of the term ‘Not breathing’ increased the likelihood of call handlers’ recognition of OHCA.

**Table 3:** Adjusted odds ratio in a model for recognition of OHCA by call handlers using key indicator symptoms

Key indicator symptom and demographic:	OR	95%CI Lower	Upper	***p***-value
Breathing Yes/Effective	0.29	0.13	0.65	0.003
Not Breathing	3.76	1.69	8.41	0.001
Abnormal Pulse/Heart Rate	0.26	0.03	2.01	0.197
Reduced or Fluctuating Consciousness Level	0.24	0.09	0.63	0.003
Female	0.40	0.17	0.91	0.029
Age 65 and over	1.78	0.80	3.95	0.157

## Discussion

This study identified the key indicator symptoms used by callers which are associated with OHCA and developed an adjusted model of symptoms for call handlers’ recognition. The caller reporting ‘Not breathing’ substantially increased the likelihood of correct EMS dispatch in OHCA, whereas the reporting of any of: ‘Breathing yes/effective’; ‘Abnormal pulse or heart rate’; ‘Reduced or fluctuating conscious level’, or the patient being female, decreased the likelihood of a genuine OHCA call being correctly dispatched.

This study is one of few about OHCA indicator symptoms where audio recordings of EMS calls have been directly analysed, rather than relying solely on EMS call documentation. A previous study, in which the words used by callers to describe the emergency were reviewed and analysed, involved listening only to calls classified as suspected CA [12]. Our study is the first to use this methodology with a comprehensive sample comprising TP, FP, FN, and TN calls to estimate sensitivity and specificity of key indicator symptoms. Of 18 previous studies which reported the sensitivity of recognition of OHCA in adults, eight (one from the UK) were of systems utilising AMPDS, and two were of similar systems [17, 25]. Call handler sensitivity (72.8%) and specificity (99.4%) for OHCA in our study were similar to previous studies elsewhere.

For OHCA the symptoms reported most frequently were unconsciousness, not breathing and ineffective breathing. This is in line with both the 2010 [26] and 2015 [27] European resuscitation guidelines, which emphasise the importance of recognising agonal/abnormal breathing as well as absence of breathing in combination with unresponsiveness. A wide variety of terms was used by callers to describe conscious level, which has also been found in EMS calls in acute stroke [28]. Likewise, as in previous studies [12, 29] the key indicator symptom of ineffective breathing encompassed a range of terms such as ‘funny breathing’ and ‘gasping’. Other terms which may be helpful in identifying OHCA, found in this and other studies, included changes in colour or coldness to touch [11, 12].

In a systematic review of 23 studies, including four which analysed the actual words used by callers, the symptoms most commonly reported during calls for OHCA were a combination of unconsciousness and absence of breathing or presence of abnormal breathing, with reported sensitivities ranging from 38 to 97% [9]. These findings are in keeping with the present study, where sensitivity of the key indicator symptom ‘unconscious’ was 86.0%, and sensitivity of ‘ineffective breathing’ was 57.3%. Only two callers in the present study used the term ‘cardiac arrest’ or a synonym; previous research has reported similarly low rates (< 4%) [29].

OHCAs in women were less often correctly recognised by call handlers than they were in men. Call handler under-recognition of OHCA in women has not been previously reported, and the reasons for it are unclear, but it may be a contributory factor to the lower rates of bystander CPR and survival reported by a recent study in another setting [30].

Current guidelines and AMPDS protocols are consistent with the most commonly reported symptoms of OHCA: unconsciousness, not breathing and ineffective breathing [19, 27]. Despite refinement of dispatch protocols over the past decade, there has been little improvement in practice and outcomes in pre-arrival recognition of OHCA by EMS. In our study, there would have been a 7% improvement in sensitivity (from 72.8 to 79.8%) if there had been complete fidelity to the current dispatch protocol. However, any changes in practice could also reduce specificity: in our study, complete fidelity to the dispatch protocol would reduce specificity from 99.4 to 95.7%, leading to more ‘false positive’ OHCA calls, and this might adversely affect overall EMS performance due to the misallocation of resources to these less urgent calls. At the time of the study, call handlers were required to select a dispatch code within 60 s, but recent changes allow for this process to take longer in calls that initially appear to be less urgent. It is apparent from the high proportion of FN cases where CPR instructions were then given by the call handler (24%) that the 60-s target might not allow sufficient time for optimum selection of a dispatch code. Call handlers in these cases clearly recognised later in the call that cardiac arrest had occurred, and if these cases were reclassified as TP cases, this would give a sensitivity for call handler recognition (as opposed to correct dispatch alone) of OHCA of 79.3%, matching sensitivity for complete fidelity to the dispatch protocol. There are inherent challenges in recognising OHCA over the telephone, with information which is often limited, and from a distressed caller, making it difficult to achieve significant improvements in this recognition rate, but targeted training for EMS call handlers, including agonal breathing, interrogation strategy, simulation training, structured dispatcher feedback, and training on telephone CPR, has been found to be effective [31]. It is also important, however, that individual services continue to benchmark against comparable ones with the highest detection rates and examine where their practice might be improved or maintained.

Overall rates of bystander-initiated resuscitation in OHCA were low (10.4%), and whilst an additional 40% of cases received CPR instructions, this still leaves half of OHCAs where no resuscitation took place before the arrival of ambulance personnel. In some cases, this appeared to be due to the caller having difficulty in carrying out essential preliminary first aid measures such as positioning the patient, and checking and clearing their airway. Achievement of improvement on these figures is challenging given the limited long-term effectiveness of public education campaigns. However, such campaigns might also indirectly facilitate callers to follow CPR instructions more quickly and effectively in those cases where bystander resuscitation has not already been commenced. Even when CPR does take place, there may be delays to its commencement which reduce the chance of survival. An earlier study found that the median delay from the start of the EMS call and commencement of tCPR was 5 min 28 s, over half of this time being taken up with assessment of breathing and other clinical signs, even though there was sufficient information to be highly concerned that the patient was in cardiac arrest within one minute of the start of the call in 71% of cases [32]. Adherence to EMS call protocols must not be at the expense of swift recognition of OHCA.

Further research including the development and testing of the effectiveness of an evidence-based training package for call handlers, audit and feedback could be undertaken. There is also early evidence that machine learning algorithms could provide automated support for call handlers to reduce the chance of a trigger cardiac arrest symptom being missed [33]. More detailed exploration of public understanding of common key indicator symptoms and their significance may lead to refinements in current protocols to use questions which are better understood by callers. The poorer recognition of OHCA in female patients is an unexplained finding which merits detailed analysis of caller-call handler dialogues. Finally, any further research should include all OHCA patients, not just those who are transported to hospital by EMS.

### Limitations

Our findings may not be generalizable to other services within or outside the UK, especially where systems other than AMPDS are used, or where the role and training of EMS call handlers differs. Our definition of OHCA included only events occurring before arrival of EMS on scene, rather than those arising *en route* to hospital. The study included only events where the patient was conveyed to the study hospital, as funding for this work precluded analysis of non-conveyed patients including those categorised as Recognition Of Life Extinct, whether they died on-scene or during conveyance to hospital. Such patients, who account for around 63% of all OHCA incidents [34] might present differently from those included in this study and the predictive value of key indicator symptoms might therefore be different if this wider patient group had been included. The clinical presentations of those patients who are not transported might include a higher proportion of incidents where the patient has arrested due to an irreversible underlying condition rather than a potentially reversible cardiac event, unwitnessed events, or those where the cardiac arrest has occurred some time before the call is made. This study included six paediatric cases, but their inclusion is unlikely to impact on the reported conclusions.

## Conclusion

There are a small proportion of calls to EMS in which cardiac arrest indicators are described but the call is not dispatched as such. Stricter adherence to dispatch protocols may improve OHCA recognition by call handlers; this should be reflected in basic and ongoing call handler training. However, the existing dispatch protocol would not be improved by the addition of further terms: this would be at the expense of dispatch specificity and would increase the number of ‘false positive’ OHCA dispatches, with consequent detrimental effects on overall EMS performance.

## Supplementary Information

**Additional file 1: Supplementary Table 1: T**he twelve counts generated by the complex DTA design for a reported symptom. **Supplementary Table 2:** The two 2x2 marginal tables obtained by summation of the observed counts. **Supplementary Table 3:** Unadjusted odds ratio (OR) for key indicator symptoms.

## Data Availability

The datasets generated and analysed during this study are not publicly available due to privacy reasons but are available from the corresponding author (JME Gibson) on reasonable request.

## Abbreviations

AMPDS: Advanced Medical Priority Dispatch System
CA: Cardiac arrest
CPR: Cardiopulmonary resuscitation
DTA: Diagnostic test accuracy
EMS: Emergency medical services
EPRF: Emergency medical services Patient Report System
FN: False negative
FP: False positive
OHCA: Out of hospital cardiac arrest
TN: True negative
TP: True positive
