# Supplementary material for: Predictors of recognition of out of hospital cardiac arrest by emergency medical services call handlers in England: a mixed methods diagnostic accuracy study
Source: Scand J Trauma Resusc Emerg Med. 2021 Jan 6;29:7. doi: 10.1186/s13049-020-00823-9 (PMC7789721; doi:10.1186/s13049-020-00823-9)
Supplement: Supplementary file 1 — Additional file 1: Supplementary Table 1: The twelve counts generated by the complex DTA design for a reported symptom. Supplementary Table 2: The two 2x2 marginal tables obtained by summation of the observed counts. Supplementary Table 3: Unadjusted odds ratio (OR) for key indicator symptoms. [file 13049_2020_823_MOESM1_ESM.docx]

Supplementary file for manuscript STRE-D-20-00205

*Data Analysis*

The counts shown in Supplementary Table 1 were collapsed by summation to form two 2x2 marginal tables for computation of the DTA measures by the standard formulae (Supplementary Table 2), namely

Sensitivity = 100a/(a+b), Specificity = 100d/(c+d), PPV = 100a/(a+c), NPV = 100d/(b+d) where a, b, c and d are defined in Table 2. The first marginal table was for OHCA (present or absent) by call handlers dispatch (positive or negative). This provided an estimate of test accuracy for call handlers’ recognition of OHCA. The second marginal table was for OHCA (present or absent) by symptom/s reported (positive or negative). This provided estimates of test accuracy for recognition of OHCA by a reported symptom or combination of symptoms. In forming the marginal tables, the sampling fraction was used to inflate the observed TN counts to adjust for the sampling of the true negatives at step 3.

| **Supplementary Table 1: The twelve counts generated by the complex DTA design for a reported symptom** | | | | | |
| --- | --- | --- | --- | --- | --- |
|  |  | Diagnostic tests: | | | |
|  |  | Call handlers’ dispatch positive for CA | | Call handlers’ dispatch negative for CA | |
| Gold standard:  Hospital and EMS records | | Symptom/s reported | Symptom/s not reported | Symptom/s reported | Symptom/s not reported |
| CA present | OHCA present | C4 | C10 | C3 | C9 |
|  | OHCA absent | C5 | C11 | C2 | C8 |
| CA absent | OHCA absent | C6 | C12^2^ | C1^1^ | C7^1^ |

^1^ Counts from the sample of calls; for computation of DTA measures, these counts are divided by the sampling fraction 0.0128.

^2^C7 to C12 are obtained by subtracting the corresponding count of symptoms reported from the number of calls listened to.

| **Supplementary Table 2: The two 2x2 marginal tables obtained by summation of the observed counts** | | | |
| --- | --- | --- | --- |
| **Recognition of OHCA by call handlers** | | | |
|  |  | Call handlers dispatch positive for CA | Call handlers dispatch negative for CA |
|  | OHCA present | a = C4+C10 | b = C3+C9 |
|  | OHCA absent | c = C5+C6+C11+C12 | d= (C1/f) +C2+(C7/f) +C8 |
|  | | | |
| **Recognition of OHCA by reported symptom/s** | | | |
|  |  | Symptom/s reported | Symptom/s not reported |
|  | OHCA present | a = C3+C4 | b = C9+C10 |
|  | OHCA absent | c = (C1/f) +C2+C5+C6 | d = (C7/f) +C8+C11+C12 |

The inclusion of the sampling fraction in the formulae had the consequence that standard confidence interval formulae would not account for the additional error introduced due to the sampling of the TN cases. Also, in one of the two marginal 2x2 tables, the sampling fraction appeared in more than one cell of the table. For this reason, bootstrap confidence intervals were obtained. For each symptom, the voice calls were tagged using their Table 1 cell membership. From the tagged voice calls, 6000 bootstrap samples were drawn and Table 1 was redrawn for each of these and the formulae for the DTA measures were applied to obtain 6000 bootstrap point estimates of the measures. Bias corrected and accelerated 95% confidence intervals were obtained from the bootstrap estimates. This methodology thus took account of the additional error introduced by the sampling of the TN cases. It also allowed the point estimates of the DTA measures to be assessed for bias; these were found to be negligible.

**Supplementary Table 3: Unadjusted odds ratio (OR) for key indicator symptoms.**

| **Key indicator symptom:** | **OR** | **95%CI**  **Lower** | **Upper** | **p-value** |
| --- | --- | --- | --- | --- |
| Collapse | 0.63 | 0.32 | 1.23 | 0.174 |
| Change in Colour | 1.09 | 0.52 | 2.29 | 0.816 |
| Sudden Onset/Deterioration | 0.42 | 0.17 | 1.03 | 0.059 |
| Drug/Alcohol use | NA^1^ |  |  |  |
| Died | 0.85 | 0.38 | 1.88 | 0.686 |
| Eyes open/Staring | 0.84 | 0.34 | 2065^2^ | 0.705 |
| Bystander resuscitation/Airway Management /Defibrillation use | 1.79 | 0.38 | 8.48 | 0.464 |
| Mouth/Vomit | 1.10 | 0.46 | 2.65 | 0.832 |
| Uncoordinated Movement | 1.79 | 0.38 | 8.48 | 0.464 |
| Seizure-like Activity | 0.76 | 0.22 | 2.60 | 0.665 |
| Psychiatric Symptoms | NA^1^ |  |  |  |
| Cool/Clammy/Cold | 0.84 | 0.34 | 2.07 | 0.705 |
| Major Trauma/Haemorrhage | 0.42 | 0.17 | 1.03 | 0.059 |
| Unknown or Non-specific Problem | 0.68 | 0.16 | 2.83 | 0.593 |
| Breathing Yes/Effective | 0.28 | 0.14 | 0.56 | <0.001 |
| Not Breathing | 4.78 | 2.32 | 9.88 | <0.001 |
| Ineffective Breathing | 0.74 | 0.37 | 1.47 | 0.382 |
| Noisy Breathing | 1.90 | 0.81 | 4.45 | 0.139 |
| Unconscious | 1.78 | 0.73 | 4.36 | 0.208 |
| Conscious | 0.35 | 0.15 | 0.79 | 0.012 |
| Serious/Urgent Problem | 0.89 | 0.30 | 2.64 | 0.831 |
| Death Imminent | NA^1^ |  |  |  |
| Abnormal Pulse/Heart Rate | 0.33 | 0.06 | 1.70 | 0.185 |
| No Pulse/Output | 1.98 | 0.42 | 9.30 | 0.385 |
| Cardiac Symptoms/Disease | 4.88 | 0.62 | 38.35 | 0.132 |
| Stroke-like Symptoms | 0.51 | 0.08 | 3.14 | 0.466 |
| Self-harm | 0.34 | 0.05 | 2.46 | 0.282 |
| Reduced or Fluctuating Consciousness Level | 0.25 | 0.11 | 0.58 | 0.001 |
| Female | 0.61 | 0.31 | 1.20 | 0.156 |
| Age 65 and over | 1.55 | 0.80 | 2.99 | 0.192 |

^1^ Where the OR for the key indicator symptom is shown as NA (not available), this is due to a rarely mentioned key indicator symptoms which provided identical prediction for all cases in which the symptom was heard.

^2^ This figure demonstrates that the odds ratio is estimated very imprecisely due to very small numbers contributing to estimation of the odds ratio.
